# Social determinants of tobacco consumption among Nepalese men: findings from Nepal Demographic and Health Survey 2011

**DOI:** 10.1186/1477-7517-10-40

**Published:** 2013-12-20

**Authors:** Vishnu Khanal, Mandira Adhikari, Sujan Karki

**Affiliations:** 1 Sanjeevani College of Medical Sciences, Butwal, Nepal; 2 Population Services International, Nawalparasi, Nepal; 3 UNICEF MICS, UNICEF Nepal, Kathmandu, Nepal; 4 School of Public Health, Curtin University, Perth, Australia

**Keywords:** Demographic and Health Survey, Nepal, Prevalence, Social determinants, Tobacco

## Abstract

**Background:**

In the 20th century, 100 million people across the globe lost their lives due to consumption of tobacco. Every year 15,000 deaths in Nepal are attributable to tobacco smoking and using other products of tobacco. This study aimed to establish the proportion and the social determinants of tobacco use among Nepalese men based on the Nepal Demographic and Health Survey (NDHS), 2011.

**Methods:**

This study used the NDHS 2011 data. The prevalence of cigarette smoking, other forms of tobacco 16 smoking and use of tobacco in any form is reported as a percentage (%). The significance of association of the statistically significant variables established using Chi-square test was further tested by using multiple logistic regression.

**Results:**

Of the 4121 participants, the prevalence of consuming any form of tobacco was 51.9% [95% confidence interval (CI) (49.6%- 54.3%)]; chewing/sniffing tobacco was 34.8% (95% CI: 32.4%- 37.3%) and tobacco smoking was 33.6% (95% CI 31.3%-36.0%).

Men with no education [Odds Ratio (OR) 3.477; 95% CI (2.380-5.080)], from an older age group (36–49) [OR 2.399; 95% CI (1.858-3.096)] who were from a manual occupation [OR 1.538; 95% CI (1.188-1.985)], who were married[OR 1.938; 95% CI ( 1.552-2.420)], and who were from the Terai region [OR 1.351; 95% CI (1.083-1.684)] were more likely to consume tobacco. Men who watched television at least once a week [OR 0.642; 95% CI (0.504-0.819)] were less likely to consume tobacco.

**Conclusions:**

The current study showed that over half of Nepalese men consume tobacco. There is an urgent need to fully implement Nepal’s Tobacco Control and Regulation Act which will ban smoking in public places; enforced plain packaging and display of health warnings over 75% of the packaging, and has banned selling of tobacco products to those under 18 years of age. There is a need to increase the social unacceptability of tobacco in Nepal by raising awareness through different electronic and cultural media. Anti-tobacco campaigns should focus on those who are less educated, have manual occupations, are in poorer economic groups, and are from the Terai region of Nepal.

## Background

Annually 4.9 million people in worldwide lose their lives as a result of tobacco consumption [1]. In the 20th century, 100 million people across the globe lost their lives due to consumption of tobacco [2,3]. Mathers and Loncar [4] estimated that deaths due to tobacco consumption are on the rise, from 5.4 million in 2005 to 6.4 million in 2015 and 8.3 million in 2030. Annually, tobacco is reponsible for 1.4 million cancer deaths. Lung, oral, and nasopharyngeal cancers are some of the major cancers caused by tobacco consumption [1,5]. Chronic diseases due to cigarette smoking, elevated risk of cardiovascular disease, diabetes and respiratory diseases are also the consequences of tobacco use [6,7]. The use of tobacco adds a burden to the national economy by increasing costs in health expenditure and other indirect costs related to illness due to tobacco borne diseases [1]. The World Health Organization (WHO) projected that there is an increasing trend of tobacco use in developing countries ranging from 4.9 million in 2000 to more than 10 million by 2020 [1].

The South East Asia region of the WHO alone shares the burden of 90% of global smokeless tobacco (SLT) consumers [1,8]. The overall prevalence of tobacco consumption in India was 48.9% [9]. In the South Asia region, aside from cigarrettes, many other forms of tobacco (smokeless tobacco) are consumed [9,10]. Bidi is similar to a cigarette but is handmade and has no filter. Gutka is a preparation which contain areca nut, tobacco, catechu and is flavoured with sweet; Zardapaan (betel quid) is rolled betel leaf with lime, betel nut and tobacco). Khaini is flavoured tobacco mixed with lime. Sokha is non-flavoured raw leaves of tobacco which are manually crushed, mixed with lime, and rolled in the hands before use.

Every year 15,000 deaths in Nepal are attributable to tobacco smoking and using other products of tobacco [11]. According to a recent study on Nepalese Adolescents and Youth, prevalence of tobacco smoking was reported to be 16.74% among the 15–19 year age group [12]. Based on the Nepal Demographic and Health Survey (NDHS, 2006) dataset, Sreeramareddy et al. [10] reported that the prevalence of ‘any tobacco use’ , ‘tobacco smoking’ and ‘tobacco chewing’ was 30.3%, 20.7% and 14.6%, respectively.

A number of determinants for tobacco consumption were reported. Age group, education, marital status, place of residence (region), occupation, belonging to a particular social group, and economic status of family have been frequently reported as the social determinants of tobacco use [5,10,13-16].

A number of approaches have been suggested to control tobacco use. The Government of Nepal has implemented a complete ban on tobacco advertisement on electronic and print media since the late 1990s. Recently, the Tobacco Control and Regulation Act, Nepal (2068 BS/2011) [17] was endorsed which includes: a ban on smoking in public places, enclosed houses and vehicles; displaying “No Smoking” warning signs in public places; requirements that packaging should not contain any logo, picture, or word that attracts a child (plain packaging); a ban on manufacturing goods that look similar to cigarettes, bidis or cigars; 75% of the packaging of the tobacco related products should contain warnings on the ill health effects of tobacco use and the picture of the consequences of tobacco use; a ban on adverstisement; a ban on selling or distributing free tobacco to a child aged <18 years and a pregnant mother; and a ban on the use of tobacco as a gift item. However, the implementation of these regulations and the effects of these regulations in reducing tobacco consumption are yet to be monitored. Nevertheless, the act indicates positive efforts are being made to control tobacco use in Nepal.

The former Nepalese study reported that the consumption of ‘any tobacco use’ , ‘tobacco smoking’ and ‘tobacco chewing’ was significantly higher in males than females [10]. A study on Andaman and Nicobar Islands, India reported that males were three times more nicotine dependent than females [9]. Currently there is no published research that explains the socio-demographic factors that are associated with Nepalese men who consume tobacco. Updated knowledge on tobacco use among Nepalese men who are the major consumers of tobacco products in Nepal, will provide policy makers and health managers with new knowledge to design and implement tobacco control efforts in Nepal. The current study aimed at establishing the prevalence and the social determinants of tobacco use among Nepalese men based on the Nepal Demographic and Health Survey, 2011.

## Methods

### Data source

This study used data from the Nepal Demographic and Health Survey 2011 [18]. The NDHS is a cross-sectional survey conducted every five years to report the demographic and health related characteristics of the population. This study used a two stage cluster design. In the first stage, primary sampling units (PSU) were identified based on probability proportionate to size. A total of 194 rural and 95 urban PSU were selected. Nepal is predominantly rural, therefore, the number of urban PSUs was inflated to obtain an adequate sample. PSUs are wards or group of wards in rural, and sub-wards in urban areas. Village development committees and municipalities are the smallest administrative units in rural and urban areas of Nepal, respectively. These units are further divided into wards. The wards in municipalities are highly populated, therefore, the NDHS considered sub wards (a further division of wards) in municipalities (urban areas) as equivalent to PSUs of rural areas [18]. In the second stage, households were selected based on systematic random sampling. In the NDHS, the households were the sample. From each household, the household head, and women (15–49 years) were interviewed. Men aged 15–49 were interviewed in every third household.

There were 13 domains in the NDHS 2011 for the indicator estimation [18]. These 13 domains were used for sampling design. Twelve domains were divided into urban and rural. There was only rural area in one domain (Western Mountain). Altogether there were 25 sampling domains. Since the survey used probability proportionate to size (PPS) method to sample the cluster, the sample weight was given to the specific domain. The different sampling weights were given to each strata after calculation of sampling weights [18,19].

The NDHS used three sets of questionnaires to collect the information - household questionnaire, women’s questionnaire, and men’s questionnaire. The household questionnaire was used to interview the person who reported to be the head of the house. The women’s questionnaire was used to obtain the information about the women and her child which mainly provides information on maternal and child health indicators and their socio-demographic characteristics. The men’s questionnaire included the social and demographic characteristics of men, their family planning knowledge and behaviour, knowledge on HIV and sexually transmitted infections, use of tobacco and related products. Separate datasets, for example, the children data, the individual data, and the men’s data were created by merging the information from these three questionnaires. These datasets were made publicly available to the researchers for further analysis [19]. The current study used the men’s dataset from NDHS 2011 [20].

### Variables

#### Outcome variables

The NDHS 2011 [18] asked the following questions to determine the status of smoking or the use of tobacco among the men:

1) Do you currently smoke cigarettes? (Yes/No)

2) Do you currently smoke or use any (other) type of tobacco? (Yes/No)

3) What (other) type of tobacco do you currently smoke or use? [Multiple response: Pipe/bidi/chewing tobacco/snuffs/others)

The categorisation of outcome variables were based on a previously reported study from Nepal based on the 2006 NDHS [10]. “Smokes any form of tobacco” in this study is defined as any person consuming cigarette/pipe/bidi/other smoking forms which were obtained from the responses to question 1 and 2 above. If a respondent answers yes to snuffs/chewing as a response to question 2 to any other type of tobacco form, he is categorised as “Chews/snuffs tobacco”. In third step, the final outcome variables was created (the main outcome variable of this study) as “Consumes tobacco in any form” which included either of “Smokes any form of tobacco” and “ Chews/snuffs tobacco”.

### Independent variables

We selected independent variables based on previously published NDHS based studies [10,21] and similar studies [5], and grouped the explanatory variable to three major levels-demographic, sociocultural, spatial and access to information. The demographic factors were age of respondents (15–24; 25–35 and 36–49 years); and marital status (married/defacto; single/divorced/separated). Sociocultural factors were education (no education; primary; secondary; higher levels); occupation (agriculture; professonal/clerical/service; manual skilled/unskilled; not working/not specified); religion (Hindu; others - Christian, Muslim, Buddhism, Kirat) and wealth quintile (poorest, poor, average/middle, rich, richest). Spatial factors included the places where the person belonged to - place of residence (rural; urban), development region (Eastern, Central, Western, Mid-western, Far-western), and ecological region (Mountain, Hill, Terai). Access to information included the frequency of reading newspapers, watching television and listening to the radio. Ethnicity was categorised into three categories: advantaged (Brahmin, Chhetri, Sanyasi, Gurung, Newar); disadvantaged (Janjati); and disadvantaged (Dalit). Of all these, Dalit ethnic groups are the most disadvantaged in Nepal. Ethnicity is a unique system in Nepal and is categorised based on previously published literature [21-23]. There are more than 120 castes and languages in Nepal. Analysis of this caste-based data is tedious and complex. Therefore, the castes were grouped into ethnic groups based on their similarity in terms of their socioeconomic characteristics. Development region is the administrative division of the country which includes the north to south sections of the country [18]. Ecological region is the division of country based on altitude and climate. The southern plain area bordering India is called Terai, north to the Terai is covered with forest and middle range of mountain is called Hill and the northernmost part bordering to Tibet which has high altitude mountains covered with snow is called the Mountain region.

### Statistical analysis

We presented the prevalence of tobacco use, smoking and chewing/sniffing tobacco with their 95% confidence interval (CI) as the frequency distribution. The association of independent variables with the use of tobacco is tested by using Chi square test. The significance of association of the statistically significant variables was further tested by using logistic regression. The backward elimination proceedure was used in multiple logistic regression. A p-value <0.05 is considered statistically significant. All the analyses used the Complex Sample Analysis Method to account for survey design and sample weight [21,24]. All statistical analyses were conducted using IBM SPSS Statistics for Windows, Version 19.0 (IBM Corp. Released 2010. Armonk, NY: IBM CorpUSA). The NDHS 2011 obtained ethics approval from the Nepal Health Research Council and the ethics review board of ORC Macro International. The publicly available data were de-identified.

## Results

### Characteristics of respondents

A total number of 4121 men were included in the study, 41.3% were from the 15–24 years age group followed by 30.1% in the 36–49 years age group. Six out of ten were married/in a de-facto relationship. Slightly more than half (51.9%) had completed secondary level education. Regarding occupation, 32.2% were professional workers followed by agriculture workers (31.6%). Almost half (52.3%) of the respondents were from the advantaged group followed by 35.8% from the disadvantaged Janjatis. The majority (84.6%) belonged to the Hindu religion. In response to economic status, slightly more than one quarter (27.5%) were from the richest families. Most (67.2%) of the respondents were from rural areas. Less than a half (46.6%) of the respondents were from Terai. In response to information access, 34.0% read the newspaper, 52.6% watched television, and 59.6% listened to the radio at least once a week (Table 1).

**Table 1:** **Prevalence of tobacco use among Nepalese men by socio**-**demographic characteristics**, **Nepal Demographic and Health Survey 2011** (**N** = **4121**)

**Factor**	**Number (%)**	**Smoking n (%; 95% CI)**	**Chewing n (%; 95% CI)**	**Any tobacco n (%; 95% CI)**
**Demographic factors**				
**Age of the respondents**		P < 0.001	P < 0.001	P < 0.001
15-24	1702(41.3)	354(23.0;20.5-25.8)	212(14.0;11.5-16.9)	459(29.7;26.7-33.0)
25-35	1178(28.6)	450(37.2;33.5-41.1)	544(48.0;44.2-51.8)	746(63.8;60.4-67.1)
36-49	1241(30.1)	565(44.1;40.2-48.0)	581(49.8;46.2-53.5)	868(70.0;66.5-73.2)
**Marital status**		P < 0.001	P < 0.001	P < 0.001
Married/Defacto	2628(63.8)	1083(40.5;37.6-43.5)	1165(47.3;44.5-50.1)	1707(65.7;63.3-68.1)
Single/Separated/Divorced	1493(36.2)	286(21.5;19.0-24.2)	172(13.0;10.7-15.6)	366(27.7;24.8-30.9)
**Sociocultural factors**				
**Education**		P < 0.001	P < 0.001	P < 0.001
No education	498(12.1)	292(57.9;52.5-63.1)	275(57.8;50.5-64.7)	398(80.1;75.7-83.8)
Primary	815(19.8)	381(42.8;38.3-47.5)	395(50.0;45.8-54.1)	584(69.5;65.5-73.1)
Secondary	2139(51.9)	548(27.4;24.5-30.5)	545(27.7;25.1-30.5)	858(42.8;39.7-45.9)
Higher	669(16.2)	148(20.5;17.0-24.6)	122(18.4;14.7-22.8)	233(34.8;30.2-39.7)
**Occupation**		P < 0.001	P < 0.001	P < 0.001
Agriculture	1303(31.6)	435(33.8;30.4-37.5)	447(40.0;35.6-44.6)	673(55.2;51.3-59.1)
Professional/clerical/service	1327(32.2)	426(31.9;28.6-35.4)	419(31.6;28.8-34.5)	668(50.2;46.8-53.6)
Manual (skilled/unskilled)	951(23.1)	444(46.6;41.7-51.6)	441(48.6;43.9-53.4)	651(69.4;65.3-73.2)
Not working/not specified	540(13.1)	64(13.4;9.0-19.5)	30(5.6;3.8-8.2)	81(16.9;12.4-22.7)
**Ethnicity**		P = 0.001	P < 0.004	P < 0.001
Advantaged	2156(52.3)	653(30.4;27.5-33.5)	623(31.9;28.7-35.2)	997(48.4;45.0-51.7)
Disadvantaged (Janjati)	1475(35.8)	494(34.4;30.7-38.2)	507(35.7;32.1-39.5)	761(52.5;49.0-56.0)
Disadvantaged (Dalit)	490(11.9)	222(43.2;37.5-49.1)	207(43.3;37.1-49.6)	315(63.7;58.1-69.0)
**Religion**		P = 0.152	P = 0.132	P = 0.244
Hindu	3486(84.6)	1174(34.2;31.7-36.7)	1142(35.5;32.9-38.2)	1758(52.4;49.9-54.9)
Others	635(15.4)	195(30.4;25.8-35.4)	195(31.0;26.0-36.5)	315(49.3;44.4-54.2)
**Wealth quintile**		P < 0.001	P < 0.001	P < 0.001
Poorest	711(17.3)	316(42.6;37.0-48.3)	282(42.9;36.8-49.2)	447(64.7;59.3-69.8)
Poor	688(16.7)	253(37.4;33.2-41.8)	251(40.5;35.5-45.7)	369(56.0;51.5-60.4)
Middle	727(17.6)	273(37.8;32.5-43.5)	284(44.3;38.3-50.5)	414(59.3;53.9-64.4)
Richer	861(20.9)	244(29.1;24.5-34.1)	254(29.9;26.1-34.0)	389(45.9;40.9-51.0)
Richest	1134(27.5)	283(26.5;23.3-30.0)	266(23.4;19.6-27.8)	454(41.5;37.0-46.2)
**Spatial factors**				
**Place of residence**		P = 0.001	P < 0.001	P = <0.001
Urban	1351(32.8)	386(27.7;24.8-30.8)	381(26.9;23.4-30.7)	612(44.3;40.8-47.9)
Rural	2770(67.2)	983(34.8;32.1-37.1)	956(36.5;33.7-39.4)	1461(53.6;50.8-56.2)
**Development region**		P = 0.012	P = 0.962	P = 0.595
Eastern	978(23.7)	306(32.5;28.1-37.3)	313(34.6;30.3-39.2)	483(51.7;46.4-56.9)
Central	1002(24.3)	342(35.9;31.3-40.8)	315(34.5;29.7-39.6)	514(53.6;49.4-57.8)
Western	706(17.1)	188(26.5;22.6-30.7)	240(36.5;31.9-41.3)	335(49.2;44.5-53.9)
Mid -Western	781(19.0)	302(38.9;33.1-45.1)	270(34.4;29.3-40.0)	424(53.7;48.6-58.7)
Far-Western	654(15.9)	231(35.6;30.0-41.8)	199(33.8;26.9-41.4)	317(49.8;43.5-56.1)
**Ecological region**		P = 0.007	P < 0.001	P = 0.011
Mountain	618(15.0)	244(42.1;36.7-47.7)	161(24.4;22.5-30.6)	317(53.4;48.3-58.4)
Hill	1582(38.4)	473(30.3;27.5-33.2)	482(29.9;26.7-33.2)	763(48.3;45.4-51.2)
Terai	1921(46.6)	652(35.1;31.4-39.0)	694(39.4;35.8-43.2)	993(54.5;50.8-58.2)
**Access to information**				
**Reading newspaper or magazine**		P < 0.001	P < 0.001	P < 0.001
Not at all	1415(34.3)	617(42.7;38.7-46.80	621(47.5;43.5-51.5)	916(66.4;63.4-69.3)
Less than once a week	1304(31.6)	401(31.5;28.2-35.0)	399(36.6;30.4-37.0)	610(49.1;45.8-52.4)
At least once a week	1402(34.0)	351(25.7;22.5-29.3)	317(22.5;19.7-25.4)	547(39.1;35.4-42.8)
**Frequency of watching television**		P < 0.001	P < 0.001	P < 0.001
Not at all	759(18.4)	344(44.0;39.5-48.7)	335(47.2;42.4-51.9)	505(66.7;63.9-71.2)
Less than once a week	1193(28.9)	429(37.2;32.9-41.7)	424(41.1;37.2-45.1)	641(58.2;54.3-62.1)
At least once a week	2169(52.6)	596(28.5;26.0-31.2)	578(27.8;25.0-30.8)	927(43.9;40.9-46.9)
**Frequency of listening radio**		P = 0.003	P < 0.001	P < 0.001
Not at all	511(12.4)	204(39.4;33.9-45.1)	203(40.1;35.3-45.0)	310(61.2;56.1-66.1)
Less than once a week	1153(28.0)	409(36.9;32.4-41.7)	419(41.1;36.8-45.5)	638(58.9;54.6-63.0)
At least once a week	2457(59.6)	756(30.7;28.1-33.3)	715(30.6;28.1-33.2)	1125(46.5;43.7-49.3)

### Types and use of tobacco products

Table 2 presents the weighted estimates of the prevalence of tobacco consumption. The prevalence rate of consuming any form of tobacco was 51.9% (95% CI; 49.6%-54.3%); chewing/sniffing tobacco was 34.8% (95% CI; 32.4%-37.3%) and smoking was 33.6% (95% CI; 31.3%-36.0%). The proportion of chewable tobacco use was higher than for smoking.

**Table 2:** **Prevalence of different forms of tobacco use among Nepalese men aged 15**–**49 years**, **Nepal Demographic and Health Survey 2011** (**N** = **4121**)

**Outcomes**	**Response**	**Percentage (95% CI)**
**Forms of tobacco smoke**		
Smokes cigarette (Yes)	1233	29.8(27.8-31.9)
Smokes pipe (Yes)	33	0.5(0.2-1.0)
Smokes bidi (Yes)	34	1.0(0.6-1.6)
Smokes others (Yes)	260	6.8(5.2-8.8)
**Other forms of tobacco use**		
Chews tobacco (Yes)	1332	34.7(32.3-37.2)
Sniffs tobacco (Yes)	5	0.1(0.0-0.3)
**Outcome for current study**		
Smokes any form of tobacco (Yes)	1369	33.6(31.3-36.0)
Chews/sniffs some forms of tobacco (Yes)	1337	34.8(32.4-37.3)
Consumes tobacco in any form (Yes)	2073	51.9(49.6-54.3)

### Factors associated with consumption of tobacco among Nepalese male

Table 1 shows the findings from the Chi-square test indicating the significant factors associated with with the consumption of tobacco: smoking, chewing/sniffing, or use in any form. The findings of a further examination of associated factors by using multiple logistic regression are presented in Table 3.

**Table 3:** **Factors associated with consumption of tobacco among Nepalese men**: **adjusted odd ratios from backward elimination process**

**Factor**	**Adjusted odds ratio (95% CI)**		
	**Smoking**	**Chewing/Sniffing**	**Any form of tobacco**
**Age of the respondents**	P = 0.021	P < 0.001	P < 0.001
15-24	1.00	1.00	1.00
25-35	1.141(0.898-1.452)	3.022(2.210-4.134)	1.994(1.605-2.476)
36-49	1.463(1.103-1.941)	3.109(2.245-4.304)	2.399(1.858-3.096)
**Marital status**	P = 0.018	P < 0.001	P < 0.001
Single/Separated/Divorced	1.00	1.00	1.00
Married/Defacto	1.350(1.053-1.730)	2.084(1.551-2.779)	1.938(1.552-2.420)
**Education**	P < 0.001	P = 0.019	P < 0.001
Higher	1.00	1.00	1.00
Secondary	1.571(1.176-2.098)	1.450(1.084-1.940)	1.482(1.116-1.967)
Primary	2.422(1.712-3.427)	1.949(1.283-2.960)	2.680(1.914-3.752)
No education	4.077(2.860-5.812)	1.173(1.036-2.831)	3.477(2.380-5.080)
**Occupation**	P < 0.001	P < 0.001	P < 0.001
Professional/clerical/service	1.00	1.00	1.00
Agriculture	0.768(0.605-0.975)	0.945(0.708-1.262)	0.834(0.649-1.072)
Not working/not specified	0.487(0.282-0.841)	0.309(0.195-0.492)	0.446(0.283-0.702)
Manual (skilled/unskilled)	1.324(1.051-1.667)	1.260(0.986-1.610)	1.538(1.188-1.985)
**Ethnicity**	P = 0.875	P = 0.255	P = 0.191
Advantaged	1.00	1.00	1.00
Disadvantaged (Janjati)	0.994(0.800-1.235)	0.805(0.623-1.042)	0.859(0.695-1.061)
Disadvantaged (Dalit)	1.064(0.814-1.391)	0.924(0.695-1.228)	1.082(0.813-1.440)
**Wealth quintile**	P = 0.818	P = 0.002	P = 0.135
Richest	1.00	1.00	1.00
Richer	0.911(0.660-1.258)	1.174(0.858-1.606)	1.015(0.733-1.406)
Middle	1.079(0.768-1.514)	1.952(1.340-2.842)	1.414(0.995-2.008)
Poor	1.084(0.755-1.556)	1.805(1.224-2.662)	1.237(0.880-1.741)
Poorest	1.157(0.764-1.751)	1.911(1.206-3.028)	1.534(0.984-2.390)
**Place of residence**	P = 0.146	P = 0.605	P = 0.876
Urban	1.00	1.00	1.00
Rural	1.118(0.938-1.529)	0.934(0.721-1.210)	1.019(0.800-1.298)
**Ecological region**	P = 0.092	P < 0.001	P = 0.013
Hill	1.00	1.00	1.00
Mountain	1.130(1.032-1.844)	0.556(0.424-0.730)	0.952(0.734-1.236)
Terai	1.129(0.895-1.423)	1.785(1.411-2.259)	1.351(1.083-1.684)
**Development Region**	P = 0.019	Not in Model	Not in Model
Eastern	0.953(0.682-1.332)		
Central	1.00		
Western	0.681(0.495-0.938)		
Mid western	1.092(0.778-1.534)		
Far western	1.112(0.804-1.539)		
**Reading newspaper or magazine**	P = 0.610	P = 0.018	P = 0.522
At least once a week	1.089(0.813-1.459)	0.681(0.501-0.925)	0.856(0.639-1.146)
Less than once a week	1.144(0.877-1.494)	0.921(0.703-1.206)	0.961(0.750-1.230)
Not at all	1.00	1.00	1.00
**Frequency of watching television**	P = 0.464	P = 0.275	P = 0.001
At least once a week	0.854(0.643-1.135)	0.916(0.636-1.318)	0.642(0.504-0.819)
Less than once a week	0.956(0.730-1.253)	1.131(0.826-1.549)	0.931(0.724-1.199)
Not at all	1.00	1.00	1.00
**Frequency of listening radio**	P = 0.629	P = 0.246	P = 0.085
At least once a week	1.119(0.831-1.506)	0.987(0.771-1.264)	0.799(0.591-1.078)
Less than once a week	1.160(0.888-1.514)	1.155(0.884-1.509)	1.019(0.744-1.397)
Not at all	1.00	1.00	1.00

Age of respondents, marital status, education status, occupation, ecological region, and watching television were significantly associated with the use of any form of tobacco. Compared to the young age group (15–14 years), higher aged (36–49 years) individuals were more likely [OR 2.399; 95% CI (1.858-3.096)] to consume any form of tobacco than their older counter parts. Married men or men who were in de-facto relationships were more likely [OR 1.938; 95% CI (1.552-2.420)] to be using tobacco. Education exhibited a protective effect against tobacco use, showing an increase in the odds of the use of tobacco with decreasing education levels. The men with no education were more likely [OR 3.477; 95% CI (2.380-5.080)] to consume tobacco. Being from a manual occupation had an increased odds [OR 1.538; 95% CI (1.188-1.985)] of using tobacco than for persons from professional/clerical/service jobs. Men from the Terai region were more likely [OR 1.351: 95% CI (1.083-1.684)] to consume tobacco than the men from the Hill region. Watching television at least once a week was associated with lower odds [OR 0.642; 95% CI (0.504-0.819)] of using tobacco.

Most of the variables that were significantly associated with the use of any form of tobacco were associated with the use of either smoking or chewing/sniffing tobacco (Table 3). For instance, the higher age groups, being married/de-facto, having no education, and being from manual jobs were associated with higher odds of chewing/sniffing. Additionally, being from the poorest wealth quintile was associated with higher odds [OR 1.911; 95% CI (1.206-3.028)] and reading magazines at least once a week was associated with lower odds [OR 0.681; 95% CI (0.501-0.925)] of chewing/sniffing. For smoking, in addition to factors associated with any form of use, a regional difference was exhibited. As compared to men from the Central region, men from the Western region were less likely [OR 0.681; 95% CI (0.495-0.938)] to be smoking.

## Discussion

Smoking and the use of tobacco is the single major cause of non-communicable diseases. Resulting in four million deaths a year, smoking has a great risk, socially and economically [25]. The current study aimed at reporting the proportion of Nepalese men consuming tobacco during the 2011 survey. One in three (33.6%) smoked and slightly more (34.8%) used it in some other forms. While the proportion of men smoking cigarette was 29.8%, the consumption of any form of tobacco was almost twice the level (51.9%).

In our analysis it was found that cigarette smoking constituted less than half of the total tobacco consumption. It is slightly greater than India where cigarette smoking constituted only 14% of the total tobacco use [26]. The southern region of Nepal has close cultural relations with the Indians. Likewise, many Nepalese go to India in search of a job for their livelihood. The situation of tobacco consumption in India and Nepal are, therefore, comparable and the use of tobacco products in India may influence Nepal [26]. In Nepal, many small shops are established in huts which mainly sell different types of tobacco products. Jarda, Gutka, Paan Masala, Paan, etc. are some of the chewable tobacco products sold in such shops. For many people, it is a means of livelihood. Although specific studies from Nepal are scarce, the evidence from other developing countries such as Malawi and Ethiopia suggested that a remarkable number of families depend on tobacco-based income in developing countries [27]. It is easy to establish such small shops with a relatively low investment and the benefits are higher as the consumption rate of tobacco is higher. For these reasons, the current social acceptance of tobacco consumption is a major hinderance to reducing tobacco consumption in Nepal.

Sreeramreddy et al. [10] reported a higher prevalence of tobacco consumption among the residents of Hill and Mountain regions based on the NDHS 2006 dataset. However, our findings suggest that tobacco consumption was higher among the men of the Terai region. It should be noted that Sreeramreddy et al. [10] included men and women in their findings whereas we included men only in our analyses. They also reported that tobacco chewing was higher in the Terai region than in other regions and this is similar to our findings. The Terai area of Nepal has a free border with India. Hundreds of thousands of people cross the land borders everyday. Small sachets of tobacco products (Gutka, Zarda Paan, Paan Masala, dry tobacco leaves etc.) are easily available and used in the Indian cities bordering Nepal [9,10]. In the Terai region, the families offer betel quid (Paan) and Betel (Supari) to their guests as a part of the culture. The easy availability of tobacco products, use of Paan (betel quid) as a cultural practice, and higher social acceptance could be the major reasons for such higher prevalence of tobacco use in the Terai region.

Having a manual job as a major occupation was another risk factor for tobacco consumption in this study. The individuals from manual jobs are mostly from the labour class. Prabhakar et al. [28] argued that the labour class individuals might have more misconceptions about the perceived benefits of the use of tobacco; which might increase tobacco consumption. Additionally, as compared to a person who works as a professional or in office based job, a person working in a manual job is generally likely to have less education. In some office based settings, it is likely that people avoid smoking and also avoid getting noticed of using tobacco products [9].This education and environmental factor could be a reason why occupation was a determinant of tobacco consumption.

Education and the economic status of the family have been major determinants of health related behaviours. Individuals with lower education and a person of a lower economic status are vulnerable to unhealthy behaviours such as higher alcohol consumption, higher substance abuse and less access to information [9]. These individuals also may have less capacity to access and process health promotion messages. Our finding of the association of lower education and being from the poorest wealth quintile with a higher use of tobacco accords with findings from India [9] and Nepal [10]. The effect of access to information is also demonstrated by the fact that the individuals who watched television at least once a week were less likely to consume tobacco products. A number of Janswasthya Karyakram (Public Health Program) shows are broadcasted on television [29]. This could be seen as an important positive contribution of media.

Our finding suggested that individuals who were in an older age group were more likely to consume the tobacco products than the younger age group (15–24 years). Studies from Andaman and Nicobar Islands, India [9] and Ethiopia [25,27] also reported that the older aged individuals were more likely to consume tobacco products than the younger groups. The older individuals may have longer time for trial of tobacco use. Their collective experience of such trials and having a larger circle of tobacco acceptable social settings could be reasons for such age based differences in tobacco consumption [25]. This finding may also reflect that a lower numbers of individuals may have adopted smoking habits in recent years than a decade or so earlier [10]. As reported in the previous Nepalese study based on the NDHS 2006 [10], and the Ethiopian study [25], the married individuals were more likely to consume tobacco and similar was the finding of this study. However, with available information in the DHS data [18] and literature, the reason for such higher use of tobacco among the married individuals is not clear.

### Public health implication of the current research

The current study will be useful for public health authorities in a number of ways. The higher prevalence of tobacco products among Nepalese men suggests that, despite having some efforts of tobacco control in place, it has not been very effective. Therefore, there is an urgent need to fully implement the current tobacco control act and a need to increase the social unacceptablity of tobacco in Nepal [17,30]. Educating people on the ill effects of tobacco through the media is effective. As a part of health promotion program, accompanying movies/cinemas with anti smoking/anti-tobacco messages could be useful in Nepal [17]. The recent Tobacco Control and Regulation Act has made it mandatory to include 75% of packaging with health warnings and a picture showing the adverse health outcomes of tobacco consumption [17]. This legal provision must be implemented as soon as possible to reduce the burden of tobacco use [31]. People from the Terai area, with less education and of lower economic status, need to be targeted. There is a need to implement awareness campaigns that portray tobacco consumption as socially unacceptable.

### Strengths, limitations and future research

The current study has a number of strengths. This is based on a national level study which has a validated questionnaire, and a higher response rate. Our analysis has accounted for the sample weight, cluster effect and multi stage sampling, and has provided point estimates and their 95% CIs; therefore, the findings have a higher level of precision [24]. Limitation of this study includes the cross sectional nature of data which precludes from drawing causal inferences. Tobacco use, specially smoking, is sometimes associated with social stigma. Therefore, some of the individuals may under-report their smoking habits. Likewise, this study reported on the current use of tobacco, so, it may be an under-representation of overall tobacco prevalence in the male population as some of the ex-tobacco users may have relapsed afterwards. However, this study does provide social determinants which are to be focussed for future interventions. Future research is essential to assess the effective implementation of Nepal’s Tobacco Control and Regulation Act. Exploring the attitude of the managers and owners of the public places on the tobacco ban and formative research to explore the ways to effective implementation of the recently endorsed Tobacco Control and Regulation Act would be important future research in Nepal.

## Conclusion

The current study has shown that half of the Nepalese men consume tobacco. Tobacco accounted for half of all tobacco consumption. Men from lower education, lower socieconomic status, the Terai region and higher age were more likely to consume tobacco. There is an urgent need to implement in full the recently endorsed Tobacco Control and Regulation Act in Nepal which has highlighted the major tobacco control activities. Efforts should be focused on creating perceptions of cultural unacceptability of tobacco use - either chewing or smoking - among Nepalese people.

## Competing interest

Authors declare that they have no conflict of interest. The authors declare that there is no financial support for this study.

## Authors’ contribution

VK conceptualised the study. MA and VK performed statistical analysis. VK wrote the first draft with significant contribution of MA. SK supervised analysis and contributed in manuscript revision. All of the authors read and approved the final version of manuscript to be submitted for publication.

## Authors’ information

VK holds an MPH degree. He has been working in child health programs in Nepal for more than five years. MA is a public health worker involved in women’s health program in the Western Nepal. SK holds MPH and MA degree, and excels in surveys similar to NDHS. He is a consultant for UNICEF MICS. He has been involved in series of further analysis of NHDS datasets, and was involved in the NDHS 2006.
